# Molecular Assembly of *Clostridium botulinum* progenitor M complex of type E

**DOI:** 10.1038/srep17795

**Published:** 2015-12-07

**Authors:** Subramaniam Eswaramoorthy, Jingchuan Sun, Huilin Li, Bal Ram Singh, Subramanyam Swaminathan

**Affiliations:** 1 Biology Department, Brookhaven National Laboratory, Upton, NY 11973; 2 Botulinum Research Center, Institute of Advanced Sciences, Dartmouth, MA; 3 Department of Biochemistry and Cell Biology, Stony Brook University, Stony Brook, NY 11794.

## Abstract

*Clostridium botulinum* neurotoxin (BoNT) is released as a progenitor complex, in association with a non-toxic-non-hemagglutinin protein (NTNH) and other associated proteins. We have determined the crystal structure of M type Progenitor complex of botulinum neurotoxin E [PTC-E(M)], a heterodimer of BoNT and NTNH. The crystal structure reveals that the complex exists as a tight, interlocked heterodimer of BoNT and NTNH. The crystal structure explains the mechanism of molecular assembly of the complex and reveals several acidic clusters at the interface responsible for association at low acidic pH and disassociation at basic/neutral pH. The similarity of the general architecture between the PTC-E(M) and the previously determined PTC-A(M) strongly suggests that the progenitor M complexes of all botulinum serotypes may have similar molecular arrangement, although the neurotoxins apparently can take very different conformation when they are released from the M complex.

*Clostridium botulinum* neurotoxin (BoNT) is the most toxic substance known to mankind and no therapeutic intervention is currently available for post-exposure treatment. BoNTs block the neurotransmitter release leading to botulism, a fatal disease. Different strains of *C. botulinum* produce seven serologically distinct neurotoxins, labeled A to G, which share significant sequence homology and structural folding^1^. When food contaminated with BoNTs is ingested the toxin passes through the gastrointestinal tract, transcytosed from gut lumen into general blood circulation, binds to the presynaptic membrane of the neuronal cells and then is internalized by receptor-mediated endocytosis into nerve cells. It is then translocated by an energy and pH-dependent mechanism into the cytosol where it cleaves its target, one of the three proteins forming SNARE complex responsible for vesicle fusion and docking, blocking the release of neurotransmitters thereby causing muscular paralysis and eventual death^2^,^3^.

Botulism is mostly caused by food poisoning because of improperly canned foods. BoNTs are secreted as progenitor complexes (PTC) with a non-toxic-non-hemagglutinin protein (NTNH), and a few neurotoxin associated proteins (NAPs) with or without hemagglutinin activity (HA). Depending on the number of proteins forming the complex, three kinds of complexes, *viz*., extra large (LL), large (L) and medium (M) are formed with molecular masses (and sizes) of 900 (19S), 500 (16S) and 300 kDa (12S), respectively^4^. M complex, the minimally functional complex, consists of a neurotoxin and NTNH and has no associated NAPs, L contains at least three NAPs in addition to M complex and LL is suggested to be a dimer of the L complex through an additional molecule of Hn-33^5^. Serotype A forms all three types of complexes, B, C, and D form M and L complexes. Serotype F forms only M complex while G forms only L complex^6^. Serotype E was believed to form only M complex until the isolation of a novel L complex was reported^7^. For strains forming more than one kind of complex, all of them exist simultaneously in the same supernatant^8^,^9^.

It seems that NTNH and other proteins produced simultaneously by the bacteria with the BoNT must have important role(s) to play in the intoxication process. It is known that progenitor toxin complex protects the neurotoxin during exposure to harsh conditions found in the stomach and small intestines where it is exposed to acidic pH (2.0) and peptidases like pepsin. In spite of these harsh conditions, the toxin and other components of the complex can be detected in the general blood circulation. The idea that they play an important role is also based on data that suggest drastically-enhanced oral toxicity of the progenitor toxin compared to the purified BoNT^10^. Also, the NAPs bind to glycoproteins on the surface of the epithelial cells for transcytosis of toxin. The mechanism by which the neurotoxin is protected by the NAPs or the precise mechanism of transcytosis is not yet known. While some of the NAPs of serotypes A, B, C and D show hemagglutinin activity none of the NAPs of E and G shows any hemagglutinin activity.

Three-dimensional structures of the PTC molecular assembly are necessary to understand the mechanism by which the toxin is protected from adverse environment of gastrointestinal tract by the associated proteins and the transcytosis of toxin from epithelial membrane into general blood circulation. The crystal structure of a reconstituted progenitor M complex of type A botulinum neurotoxin has been solved, where the toxin is an inactive triple mutant^11^. Low resolution cryo-EM structures of PTC-A(L), PTC-B(L) and PTC-E(M) have also been reported^12^. Also, a reconstructed model of PTC-A(L) combining EM and individual X-ray structures has been reported^13^. Here we are reporting the X-ray structure of PTC-E(M) to understand the molecular basis for the assembly (at low pH) and disassembly at neutral and basic pH. We have used EM and X-ray crystallography to unravel the conformational changes accompanying the assembly of the complex. While the crystal structure of PTC-A(M) is made of an inactive triple mutant of BoNT/A and a recombinant NTNHA, PTC-E(M) complex used in this study is purified from clostridium culture and was fully active. The domain organization in uncomplexed BoNT/A and BoNT/E structures are drastically different raising an interesting question about their respective conformations in the complex, PTC^14^,^15^. This is the first crystal structure of an active PTC of any serotype.

## Results and Discussion

### Crystal structure of PTC-E(M) complex

**Figure 1:**
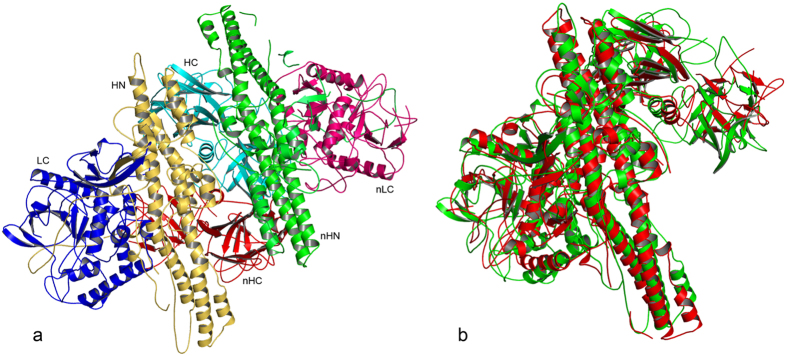
(**a**) Ribbons representation. Heterodimeric complex of PTC-E(M) is shown with each domain colored differently. (**b**) Superposition of BoNT/E and NTNHE. BoNT/E and NTNHE are shown in green and red, respectively. Both have similar fold despite low sequence homology and the overall rmsd is 7.2 Å.

The crystal structure determination of PTC-E(M) complex is described in Methods section. PTC-E(M) comprises BoNT/E holotoxin (1251aa & 144 kDa) and NTNHE (1162aa & 137 kDa) both of very similar molecular mass. PTC-E(M) crystallized in space group P3_1_ with three complexes per asymmetric unit. The final R and R-free values are 0.24 and 0.32, respectively. The quality of the structure was validated by Procheck^16^. The two molecules have a very similar fold in spite of low sequence homology (21.7% identity), each consisting of three similar domains (Fig. 1a). In this paper the three domains of BoNT/E are called LC, HN and HC corresponding to the light chain (catalytic domain), the N terminal half of the heavy chain (translocation domain) and the C terminal half of the heavy chain (receptor binding domain), respectively. The corresponding domains of NTNHE are called nLC, nHN and nHC following the convention of Gu *et al.*^11^. The two molecules form a heterodimer related by a near two fold symmetry and agree with an rmsd of 7.2 Å for 829 Cα pairs (Fig. 1b). BoNT/E is un-nicked and the disulfide bond connecting the light and heavy chain is clearly visible in the electron density map. A representative electron density region is shown in supplementary material (Supplementary Fig. S1). The three complexes in the asymmetric unit agree with an rmsd of ~0.5 Å.

**Figure 2:**
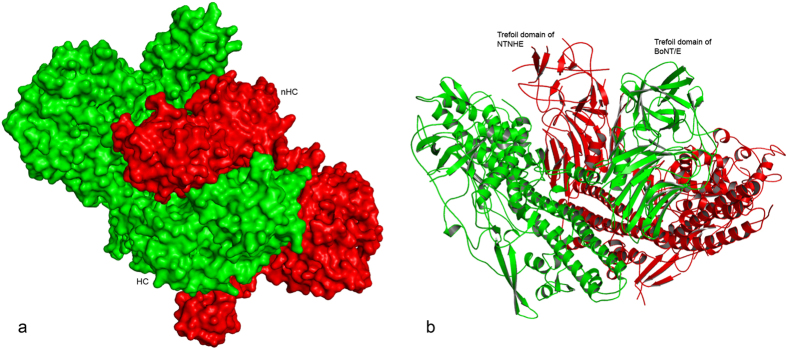
(**a**) Surface representation of PTC-E(M) complex. BoNT/E and NTNHE are shown in red and green, respectively. Binding domains (labeled as HC and nHC) are swapped to form an interlocked complex. (**b**) Trefoil regions of HC and nHC come together and point in the same direction. Color scheme is same as in (**a**).

BoNT/E and NTNHE form a tight complex with about 30255 Å^2^ buried surface area together (about a third of the total surface area of the complex). The binding domains HC and nHC face each other and are in the middle of the complex providing most of the interactions at the interface (Fig. 2a). The binding domains are swapped such that nHC is closer to LC + HN of BoNT/E and HC closer to those of NTNHE. Recently, a sequence motif (QXW) responsible for sugar binding has been identified in the trefoil fold region of NTNHE^17^. In the crystal structure of PTC-E(M), the trefoil folds of both nHC and HC come together and point in the same direction with both the sugar binding region of NTNHE and the ganglioside/protein receptor binding region of BoNT/E available for binding to glycans of the epithelial cell walls. The two glycan binding sites may act synergistically on the cell surface to promote the toxin transcytosis (Fig. 2b). The HC of BoNT/E makes contact with all three domains of NTNHE and vice versa. In summary, there are 224 non-bonded interactions (<4 Å) between the two with fifteen of them being hydrogen bond or salt bridge interaction. Also, a few acidic residues from both molecules make strong hydrogen bond interactions at the pH (<5.0) used for crystallization. As discussed later these provide the necessary interactions to keep the complex together at acidic pH. While HC, HN, nHC and nHN all interact with one another, LC and nLC are at the two extremes of the complex and do not interact. There is one salt bridge between the LC and nHC (K342 and D1149, respectively). Both LCs are exposed to solvent region. Interestingly, the active site is exposed to the solvent as in the crystal structure of BoNT/E in the uncomplexed form (hereafter referred to as BoNT/E(UC))^14^. Superposition of BoNT/A LC in complex with SNAP25 peptide^18^ shows that SNAP25 can occupy a similar site in PTC-E(M) (Supplementary Fig. S2). This may be the reason for SNAP25 being cleaved *in vitro* by PTC-E(M) when BoNT/E is in unreduced condition^19^. This is contrary to the known fact that the native BoNT/E must be reduced and nicked for SNAP25 cleavage. However, the physiological relevance of this is not clear since SNAP25 is not present in GI tract and BoNT/E is specific for neuronal SNAP25. It is suggested that BoNT/E in PTC-E(M) is in a proper conformation for SNAP25 to be cleaved without the need for reduction of disulfide bond and separation of LC from the rest of the molecule.

Although PTC-E(M) was crystallized at an acidic pH, given the known sensitivity of the complex to the buffer conditions, we asked if the crystallization mother liquor had had an influence on the interface between BoNT/E and NTNHE. We therefore determined a 17-Å resolution negative stain EM structure of the M-particle in the purification buffer of (50.0 mM MES and 100 mM NaCl – pH 5.0) (Supplementary Fig. S3). The overall size and shape was similar to a previous low-resolution EM map determined from a preparation of heterogeneous PTC-E(M) complexes^12^. By docking the PTC-E(M) crystal structure into our EM map, we found that the solution structure of the M-particle was very similar to the crystal structure, except for a minor and ~9° correlated tilt of both HC and nHC (Supplementary Fig. S3). Therefore, the interface between HC and nHC observed in the crystal structure appears to be a faithful description of the native M-particle structure.

### Both BoNT/E and NTNHE undergo conformational change when the complex associates or disassociates

**Figure 3:**
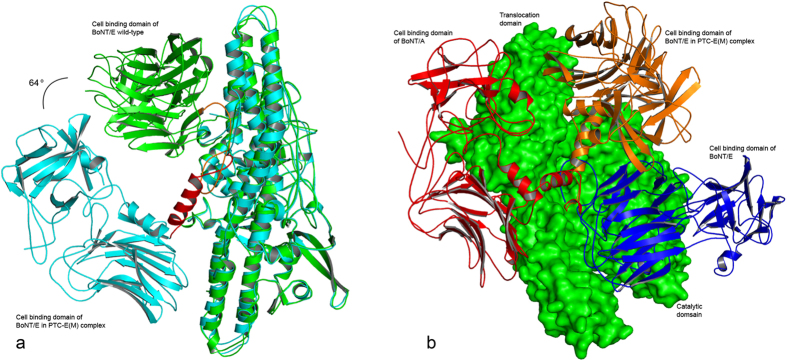
(**a**) Superposition of BoNT/E(UC) in crystal structure and in PTC-E(M) complex. The binding domain rotates about the helical linker shown in red. The binding domain rotates by ~60° on separation from the complex. (**b**) Superposition of BoNT/E in PTC-E(M) complex, BoNT/E(UC) and BoNT/A(UC). The translocation and catalytic domains are shown in surface diagram (green). The binding domains of BoNT/A (UC), BoNT/E(UC) and BoNT/E in PTC-E(M) complex are shown in ribbon representation in red, blue and orange, respectively. The linker (shown at the center of the figure) is flexible and is responsible for this variation in conformation. In BoNT/E(UC) the linker is a loop while it is a helix in the other two.

The crystal structure of BoNT/E(UC) showed a different type of domain organization compared to BoNT/A or BoNT/B and the difference is not due to the pH of crystallization or crystal packing. A flexible linker (region 830-845) connecting the HN and HC domains enables this change in conformation possible^14^. The HC (in BoNT/E) is rotated by ~120° with respect to HC of BoNT/A or B. The conformation of HC of BoNT/E in PTC is different from that of BoNT/E(UC). It rotates further by another ~60° from that of BoNT/E(UC) (Fig. 3). Presumably, when BoNT/E separates from the complex it changes its conformation to increase the domain-domain contact. Indeed, the contact surface area between the HC domain and the rest of BoNT/E increases from 2833 Å^2^ to 3848 Å^2^ and the number of interactions correspondingly increases from 115 to 165 to make the protein more stable and globular. In addition, the rotation of HC on release from the complex puts the ganglioside binding region on the same side of transmembrane region in the translocation domain (N terminal end) facilitating faster translocation of the toxin^14^.

### NTNHE is a dimer in solution

**Figure 4:**
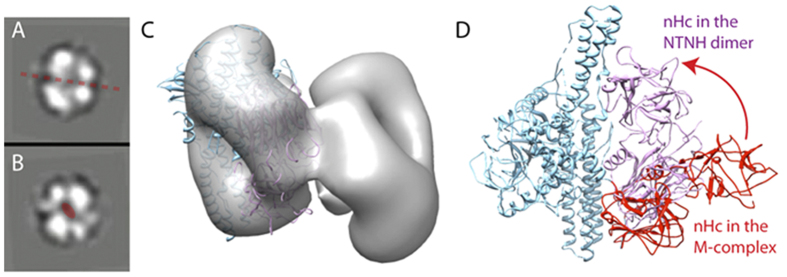
NTNHE is a dimer in solution. (**A,B**)) 2D averages of EM images (**C**) 3D map docked with one copy of NTNHE structure. (**d**) The nHC undergoes a large rotation in the M-particle.

Because the crystal structure of NTNHE alone was unknown, it was unclear whether NTNHE underwent similar structural changes upon binding with BoNT to form the M-particle. We therefore carried out EM of the purified NTNHE diluted to a concentration of ~0.05 mg/ml. Surprisingly we found that NTNHE formed a dimer in solution (Fig. 4). Some of the reference-free 2D class averages of the NTNHE EM images clearly showed mirror symmetry (Fig. 4A,B). Blue Native gel also showed that the purified NTNHE formed a dimer in solution even at a modest concentration of 1.0 mg/ml (Supplementary Fig. S4). We went on to determine a 3D reconstruction of the NTNHE dimer (Fig. 4C). We found that the conformation of NTNHE in the PTC-E(M) had to be modified in order to fit the EM density of NTNHE dimer (Fig. 4C,D). Specifically, the binding domain (nHC) had to be rotated up towards the nHN domain by ~50°.

When BoNT/E separates from the complex the binding domain of NTNHE will lose its interaction with the binding domain of BoNT/E exposing its hydrophobic regions. The EM study of the uncomplexed NTNHE shows that it forms a dimer in solution. The binding regions rotate to form a tight complex with the binding domains of the two protomers interacting. The binding domain of the other protomer of the NTNHE dimer compensates any loss of interaction with BoNT/E binding domain. Therefore, it appears that both BoNT/E and NTNHE proteins undergo drastic changes at the HC/nHC regions when forming the PTC-E(M) complex. A similar conformational change was observed in NTNHD by small angle X-ray scattering, though the rotation angle of the binding domain was different in NTNHD^20^.

### Acidic interactions responsible for tight complex formation and the separation at neutral and basic pH

PTC-E(M) complex is formed by BoNT/E and NTNHE and the complex is stable at pH 6.0 or below based on equilibrium and kinetic binding analysis of these two proteins in purified forms^21^. When the complex enters the general circulation it disassociates at neutral pH. There are 224 non–bonded interactions between the two proteins and several hydrogen bond contacts. Of special interest is the hydrogen bond interactions formed between acidic residues (Glu, Asp and His) from the two partners. We have identified six such interactions where the acidic side groups form hydrogen bond or near hydrogen bond interactions. They are BE:Asp469–NTNHE:Asp1149, BE:Glu558–NTNHE:Glu571, BE:Asp598–NTNHE:Asp954, BE:Asp817–NTNHE:Glu899, BE:Asp1013–NTNHE:Asp774 and BE:His1231–NTNHE:Glu795 (Table 1 and Supplementary Fig. S5). At the crystallization condition (pH < 5.0) these residues are most likely protonated and hence not charged. The PTC complex is supposedly intact when it resides in the gut and gets separated when they are released into general circulation at neutral or higher pH. We propose that the neutral or basic pH causes the acidic side chains to deprotonate and become negatively charged. The repulsion between the negative charges causes the two component proteins to separate, leading to the dissolution of the M complex.

**Figure 5:**
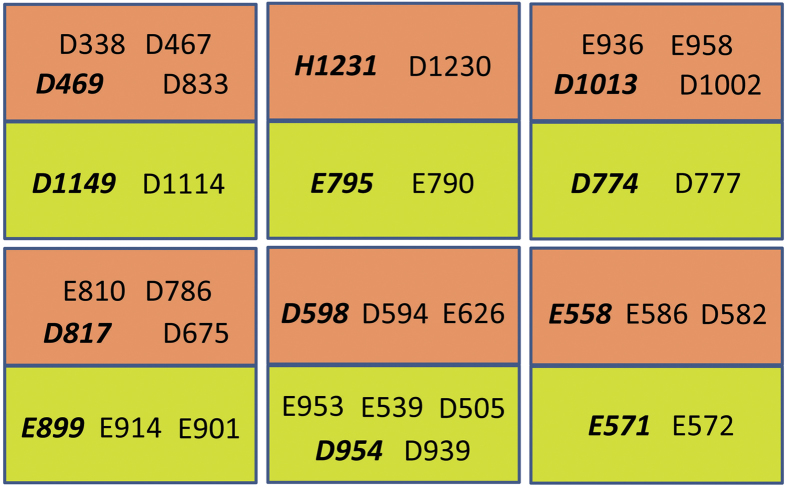
A schematic diagram showing acidic interactions at the interface. Six clusters are shown here. Residues of BoNT/E are in orange box in each cluster and residues from NTNHE are shown in green box. Residues involved in the closest acidic interactions are shown in bold italics in each cluster.

Do these acidic interactions alone act as pH sensors ?  Analysis of the interface between NTNHE and BoNT/E brings out interesting features about the interface. It is true that there are specific acid-acid interactions between the partners. But in addition, many acidic residues from both partners cluster around these specific interactions (Fig. 5). There are six such clusters as shown in the figure. Acidic residues in each cluster are within 15 Å radius. Since electrostatic forces have long-range effects, these negative charges in such close proximity increase the force of repulsion causing the partners to dissociate at neutral pH when they get deprotonated and negatively charged (Supplementary Fig. S6). We conclude that association or disassociation is not solely due to any single or a few interactions but is the sum total effect of all these repulsive forces.

### Comparison of PTC-A(M) and PTC-E(M)

Crystal structure of a reconstituted PTC-A(M) from an inactive triple mutant of BoNT/A and recombinant NTNHA has been reported^11^. BoNT/A and /E share 39% sequence identity while it is 66% between NTNHA and NTNHE. Though the crystal structures of BoNT/A(UC) and BoNT/E(UC) have distinctly different domain organization their conformations in PTC complexes are surprisingly very similar. This observation prompts us to speculate that all PTC-M complexes will likely have similar structures. While the HC of BoNT/A(UC) is rotated by 140° to superpose on that of the PTC complex, the HC of BoNT/E(UC) has to rotate by ~60° about the linker to superpose on that of PTC-E(M). The two complexes as a whole agree with an rmsd of 1.3 Å (1801 Cα pairs). BoNT/A and BoNT/E in complexes agree within 1.7 Å (940 Cα pairs). NTNHA and NTNHE agree within 0.922 Å. The binding domain of BoNT/A and BoNT/E are both surrounded by the three domains of NTNH proteins. However, in PTC-E(M) complex a strong salt bridge is formed between Lys342NZ of BoNT/E-LC and Asp1149OD2 of nHC of NTNHE (Supplementary Fig. S7). In PTC-A(M) there are no interactions between the LC of the toxin to any residue of NTNHA. It is to be noted that Lys342 is in the 350 loop which can undergo some conformational change^18^. Also, the corresponding residue in BoNT/A is a phenyalanine.

The acidic residues clustering at the interface of toxin and NTNH are mostly conserved in PTC-E(M) and PTC-A(M). Of the forty acidic residues forming the clusters in PTC-E(M), about 58% are conserved in PTC-A(M). They can be grouped into six clusters as in PTC-E(M). The loss of non-conserved acidic residues is compensated by nearby acidic residues contributing to the acidic nature of the cluster and thereby to the dissociation at neutral pH. As shown for PTC-E(M) (Fig. 5), the acidic residues at the interface within a distance of 15 Å of one another are shown in Supplementary Fig. S8. Accordingly, the dissociation mechanism of PTC-A(M) may be similar to PTC-E(M).

### The missing n-loop in NTNHE

Though NTNHA and NTNHE share 66% sequence identity, a short loop region (G116-A148) called “nloop” in NTNHA is absent in NTNHE. This region is not visible in the electron density map of PTC-A(M) may be because it is nicked or disordered in the crystal structure. It is assumed that this region would interact with the HA protein in larger complexes (L or LL). The sequence corresponding to the nloop is absent in serotypes A2, E and F and accordingly it was believed that these serotypes cannot form higher MW complex with HA proteins. However, it has been shown that BoNT/E does form an L complex^19^. The function of nloop and its importance in forming larger complexes needs further investigation.

## Conclusions

1. Though BoNT/E and BoNT/A have different domain organization in uncomplexed state they take similar conformation in PTC-A(M) and PTC-E(M) complexes suggesting all M complexes may have similar architecture. 2. BoNT/E and NTNHE have similar fold in spite of low sequence homology. 3. BoNT/E and NTNHE form a tight complex by swapping HC and nHC. 4. In the M-complex the binding domain of neurotoxin is surrounded by all three domains of NTNHE. 5. The trefoil folds of both BoNT/E and NTNHE come together and point in the same direction facilitating synergistic binding to epithelial cell. 6. A number of acidic interactions play a role in association at low pH and disassociation at neutral or higher pH. 7. There are a number of acidic clusters involving acidic residues from both BoNT/E and NTNHE at the interface. 8. Our structural analyses suggest that there may not be a single pH sensor that is responsible for the M complex disassociation; rather, we believe it is the net repulsion force between opposing acidic clusters as they are deprotonated and become charged at higher pH that drives apart BoNTE and NTNHE.

## Methods

### Handling of toxin complex

Botulinum neurotoxin is classified as Select Agent Category A by the CDC and accordingly strict compliance to CDC specifications was followed. PTC-E(M) was isolated and purified in BSL3 lab at UMASS, Dartmouth registered with CDC. Crystallization was in a BSL2 level at Brookhaven National Laboratory registered with and certified by CDC for working with Select Agent, botulinum neurotoxin.

### Preparation of PTC-E(M) and NTNHE

PTC (M) was isolated according to the method of Gimenez and Sugiyama^22^. NTNHE was purified from PTC-E(M) as described in Singh *et al.*^21^ using a DEAE-A50 chromatography column equilibrated in 0.2 M sodium phosphate buffer, pH 7.4. Purity of protein preparations was tested with sodium dodecyl sulfate-polyacrylamide gel electrophoresis.

### Crystallization, structure determination and refinement

PTC-E(M) at a concentration of 7 mg/ml in a buffer containing 25 mM MES, 100 mM NaCl and 1.0 mM glutathione (pH 6.0) was used for screening crystallization condition using commercially available crystallization screens. Long needle like crystals were obtained with 10% PEG 4000 and sodium acetate buffer at pH 4.6 as precipitant. Crystals grew slowly and were stable for nearly two weeks. Crystals were mounted in cryo loops and flash frozen in liquid nitrogen using the mother liquor augmented with 20% glycerol as cryo protectant.

X-ray diffraction data were collected at beamline X29 of National Synchrotron Light Source (NSLS), Brookhaven National Laboratory. Crystals diffracted at least to 3.0 Å resolution. Data corresponding to ф = 360° were collected at 0.5° interval to obtain redundant data. PTC-E(M) crystallized in space group P3_1_ with three PTC-E(M) complex (BoNT/E and NTNHE) per asymmetric unit and the Matthews coefficient was calculated to be 3.77 Å^3^/Da corresponding to 68% solvent content by volume. Data were processed using HKL-2000^23^. Data processing statistics and unit cell parameters are given in Table 2.

Crystal structures of BoNT/E holotoxin^14^ and the NTNHA of PTC-A(M)^11^ were used as search models to determine the structure of PTC-E(M) by the molecular replacement method. While the NTNHA molecule was used as a whole, the BoNT/E model was used as two parts in the structure solution process since it is known that the HC of BoNT is flexible. A total of three search models, i) the whole molecule of NTNHA, ii) catalytic and translocation domains of BoNT/E toxin, and iii) the binding domain of BoNT/E^11^ were used in PHASER in CCP4 suite^13^,^14^.

The solution with three molecules of BoNT/E and NTNHE complex refined well in the space group P3_1_. Rigid body refinement was carried out initially with four rigid bodies per molecule, i) catalytic and translocation domains of BoNT/E toxin, ii) catalytic and translocation domains of NTNH/A, iii) the binding domain of BoNT/E, and iv) the binding domain of NTNHE. Then the model was refined with six rigid bodies, three BoNT/E and three NTNHE molecules. Electron density map was calculated at this stage and all three complex molecules were independently checked to identify any possible dissimilarity between copies. Since no difference was found between non-crystallographic symmetry (NCS) related molecules further refinements were carried out with NCS constraints between copies. COOT and Refmac 5.7 were used for model building and refinement, respectively^24^. Refinement statistics are given in Table 2.

### EM studies of M complex and NTNHE

EM grids were prepared in a specially designated biosafety lab (BSL2). The purified M complex or NTNHE was stained in 2% uranyl acetate aqueous solution. Electron microscopy was carried out in a JEOL 2010 F TEM operated at 200 kV. Electron micrographs were recorded at a magnification of 50,000× in a 4 K by 4 K Gatan Ultrascan CCD camera. For the M-complex, we picked 10769 particles, computationally sorted the raw particle images into 100 classes in

EMAN2. Many well-defined 2D class averages were obtained. We rejected raw particle images that did not produce good class averages. After such rejection, 6657 particle images remained in the final data for 3D reconstruction. For NTNHE, we picked 10039 particles, only kept 3371 particles after reference free 2D classification. Initial model calculation and 3D refinement was performed in EMAN2, and the estimated final resolution was ~18 Å. The 3D surface rendering was prepared by UCSF Chimera.

## Additional Information

**How to cite this article**: Eswaramoorthy, S. *et al.* Molecular Assembly of *Clostridium botulinum* progenitor M complex of type E. *Sci. Rep.* **5**, 17795; doi: 10.1038/srep17795 (2015).

## Supplementary Material

Supplementary Information

## Figures and Tables

**Table 1:** Acidic interactions at the interface of PTC-E(M).

BoNT/E	NTNHE
D469	D1149
E558	E571
D598	D954
D817	E899
D1013	D774
H1231	E795

**Table 2:** Crystal Data and Refinement Parameters.

Crystal Data	
Crystal system:	Trigonal
Space group:	P3_1_
Unit cell values:	a = b = 192.60; c = 286.54 (Å)
	α = β = 90.0; γ = 120.0 (°)
Data reduction program	HKL-2000
Resolution (Å)	50.0 − 3.0 (3.11 − 3.0)
Total reflections	752,939
Unique reflections	165,586 (3345)
Completeness (%)	69.8 (14.1)
Redundancy	4.5 (1.1)
Square RSym^1^	0.24 (NA)^4^
I/Sig (I)	2.3 (NA)
Refinement Parameters	
Refinement program	REFMAC 5.7
Resolution (Å)	49.3 − 3.05
Number of reflections	133,607
R-value^2^	0.244
R-free^3^	0.321
NCS Restraint:	Local
NCS Molecules:	3
Number of atoms refined:	
Protein Chain A	9978
Protein Chain B	9005
Zn	1
All atoms:	56952
RMSD	
Bond lengths (Å)	0.011
Bond angles (°)	1.44

^1^R-fac = Σ_h_Σ_i_|I_i_(h)-<I(h)>|/Σ_h_Σ_i_ |I_i_(h)|, where I_i_(h) is the intensity measurement for a reflection h and < I(h) > is the mean intensity for this reflection.

^2^R-value = Σ_i_||F_i,obs_|-k|F_i,cal_||/Σ_i_|F_i,obs_|.

^3^R-free: Same as R-value for a subset of randomly chosen reflections.

^4^NA: Not available.
