# Supplementary material for: Molecular Assembly of Clostridium botulinum progenitor M complex of type E
Source: Sci Rep. 2015 Dec 7;5:17795. doi: 10.1038/srep17795 (PMC4671068; doi:10.1038/srep17795)
Supplement: Supplementary Information [file srep17795-s1.pdf]

Supplementary material

Molecular Assembly of *Clostridium botulinum* progenitor M complex of type E

Subramaniam Eswaramoorthy[1], Jingchuan Sun[1], Huilin Li[1,3], Bal Ram Singh[2] and Subramanyam Swaminathan[1,*]

[1]Biological, Environmental & Climate Sciences Department, Brookhaven National Laboratory, Upton, NY 11973

[2]Botulinum Research Center, Institute of Advanced Sciences, Dartmouth, MA

[3]Department of Biochemistry and Cell Biology, Stony Brook University, Stony Brook, NY 11794

[*]Corresponding author. Email swami@bnl.gov; phone: 631-344-3187

Supplementary figure legends

Figure S1. Electron density map. A representative section of the 2Fo-FC electron density map contoured at 1σ. The model could be unambiguously built in the map. However, there were regions of weak electron density where the polypeptide chain could not be traced.

Figure S2. The active site of catalytic domain is open in PTC-E(M). The BoNT/A-LC with SNAP25 peptide (1XTG) is superposed on the LC of PTC-E(M). The active site where SNAP25 binds is exposed and is accessible to SNAP25. This may be the reason that PTC-E(M) is catalytically active. BoNT/E and NTNHE of PTC-E(M) are shown in green and magenta, respectively. The BoNT/A-LC is shown in orange and SNAP peptide in dark blue. Zinc is shown as a black sphere.

Figure S3. Negative-stain EM of purified BoNT-E(M) particles. (a) A small area of the raw TEM micrograph. (b) Selected reference-free class averages of the M particles. (c and d) Rigid-body docking of the BoNT-E M-particle from the crystal structure into EM 3D map shown in two views. The BoNT/E structure is in red and NTNH-E is in cyan. The binding domains Hc/nHc appear to be slightly rotated in the crystal structure.

Figure S4. Native gel of NTNHE.

Figure S5. Six acidic interactions at the interface of BoNT/E and NTNHE in PTC-E(M) complex. 1. BoNT/E:D469 – NTNHE:D1149; 2. BoNT/E:E810 – NTNHE:E899; 3. BoNT/E:D598 – NTNHE:D954; 4. BoNT/E:E558 – NTNHE:E571; 5. BoNT/E:D1013 – NTNHE:D774; and 6. BoNT/E:H1231 – NTNHE:E571.

Figure S6. The six acidic clusters and their corresponding electrostatic potential at the interface.

Figure S7. Superposition of PTC-A(M) and PTC-E(M). Though the conformation of BoNT/A (red) and BoNT/E (green) in the wild type crystal structures are different, they have the same conformation in M complex.

Figure S8. A schematic diagram showing acidic interactions at the interface of PTC-A(M). Six clusters are shown here. Residues of BoNT/A are in orange box in each cluster and residues from NTNHA are shown in light blue box.

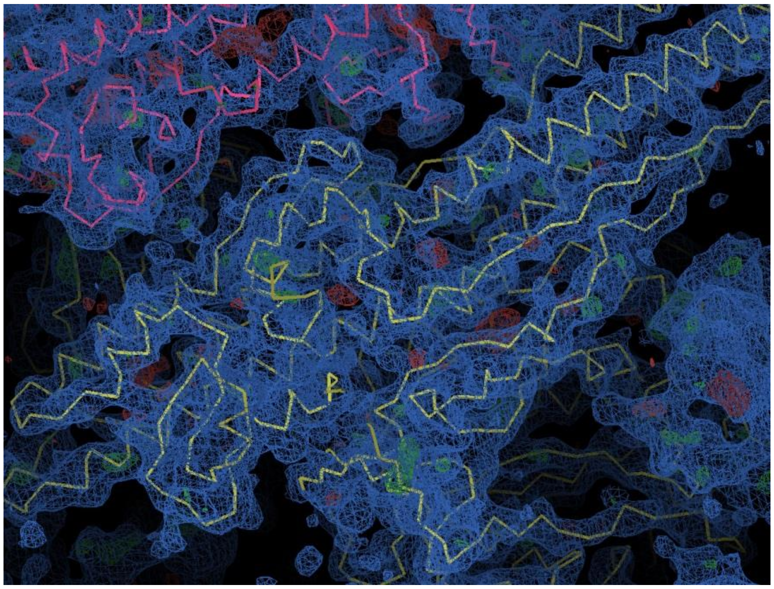

Figure S1. Electron density map. A representative section of the 2Fo-FC electron density map contoured at 1σ. The model could be unambiguously built in the map. However, there were regions of weak electron density where the polypeptide chain could not be traced.

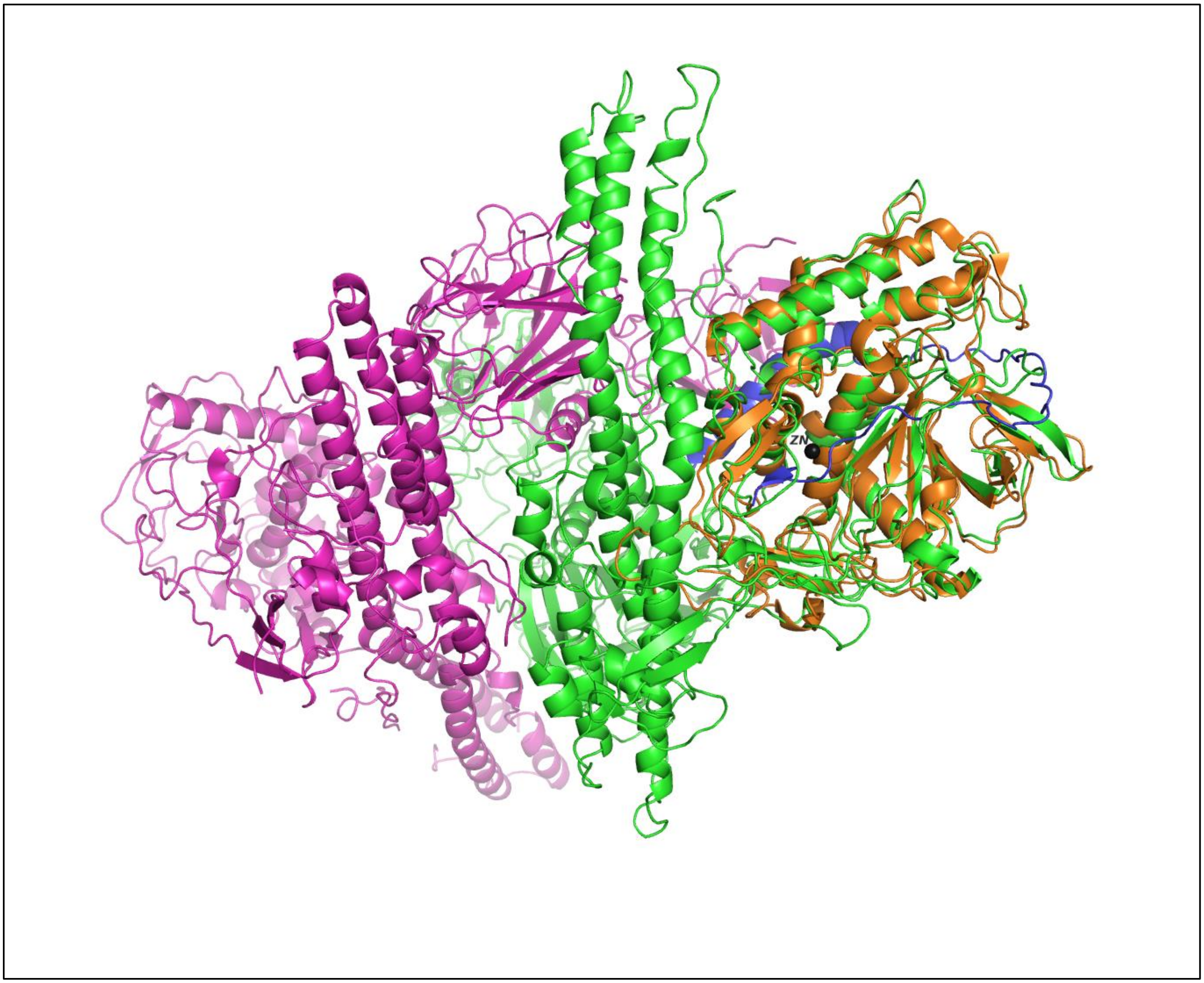

Figure S2. The active site of catalytic domain is open in PTC-E(M). The BoNT/A-LC with SNAP25 peptide (1XTG) is superposed on the LC of PTC-E(M). The active site where SNAP25 binds is exposed and is accessible to SNAP25. This may be the reason that PTC-E(M) is catalytically active. BoNT/E and NTNHE of PTC-E(M) are shown in green and magenta, respectively. The BoNT/A-LC is shown in orange and SNAP peptide in dark blue. Zinc is shown as a black sphere.

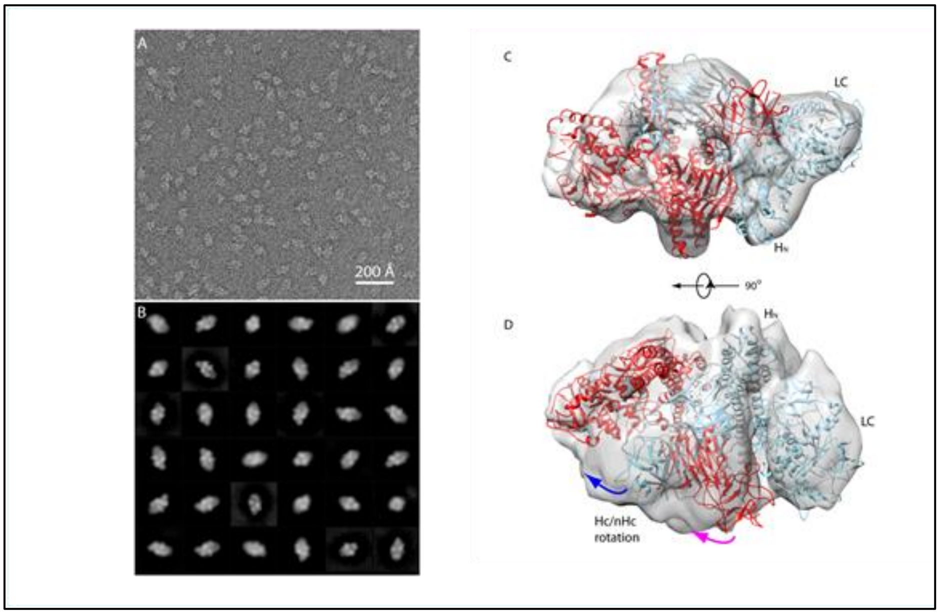

Figure S3. Negative-stain EM of purified BoNT-E(M) particles. (a) A small area of the raw TEM micrograph. (b) Selected reference-free class averages of the M particles. (c and d) Rigid-body docking of the BoNT-E M-particle from the crystal structure into EM 3D map shown in two views. The BoNT/E structure is in red and NTNH-E is in cyan. The binding domains Hc/nHc appear to be slightly rotated in the crystal structure.

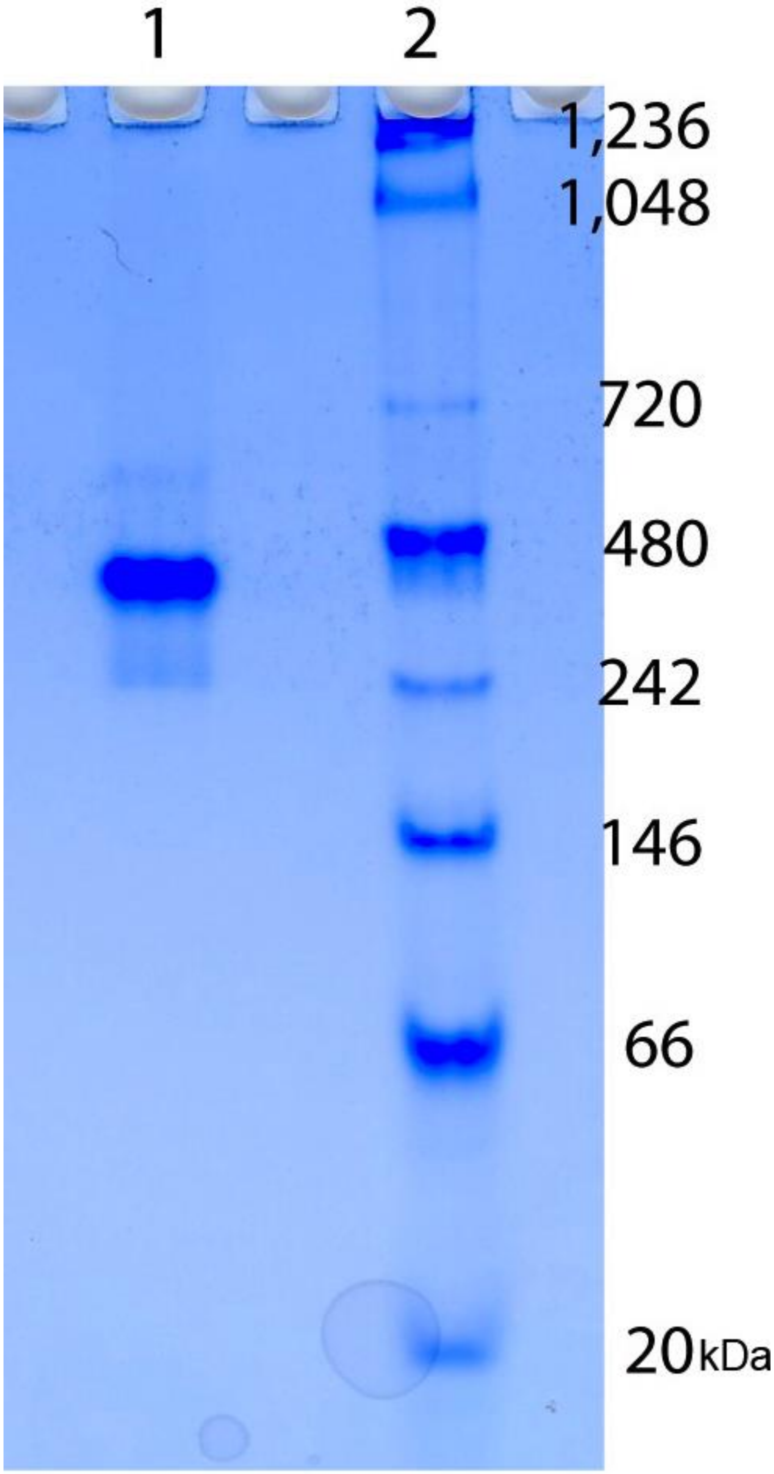

Lane 1: NTNHE; Lane 2: Protein marker

Figure S4. Native gel of NTNHE.

Figure S5. Six acidic interactions at the interface of BoNT/E and NTNHE in PTC-E(M) complex. BoNT/E and NTNHE are shown in ribbon representation in green and cyan color, respectively. BoNT/E and NTNHE residues are shown in sphere model with the carbon atoms in green and blue, respectively. 1. BoNT/E:D469 – NTNHE:D1149; 2. BoNT/E:E810 – NTNHE:E899; 3. BoNT/E:D598 – NTNHE:D954; 4. BoNT/E:E558 – NTNHE:E571; 5. BoNT/E:D1013 – NTNHE:D774; and 6. BoNT/E:H1231 – NTNHE:E571.

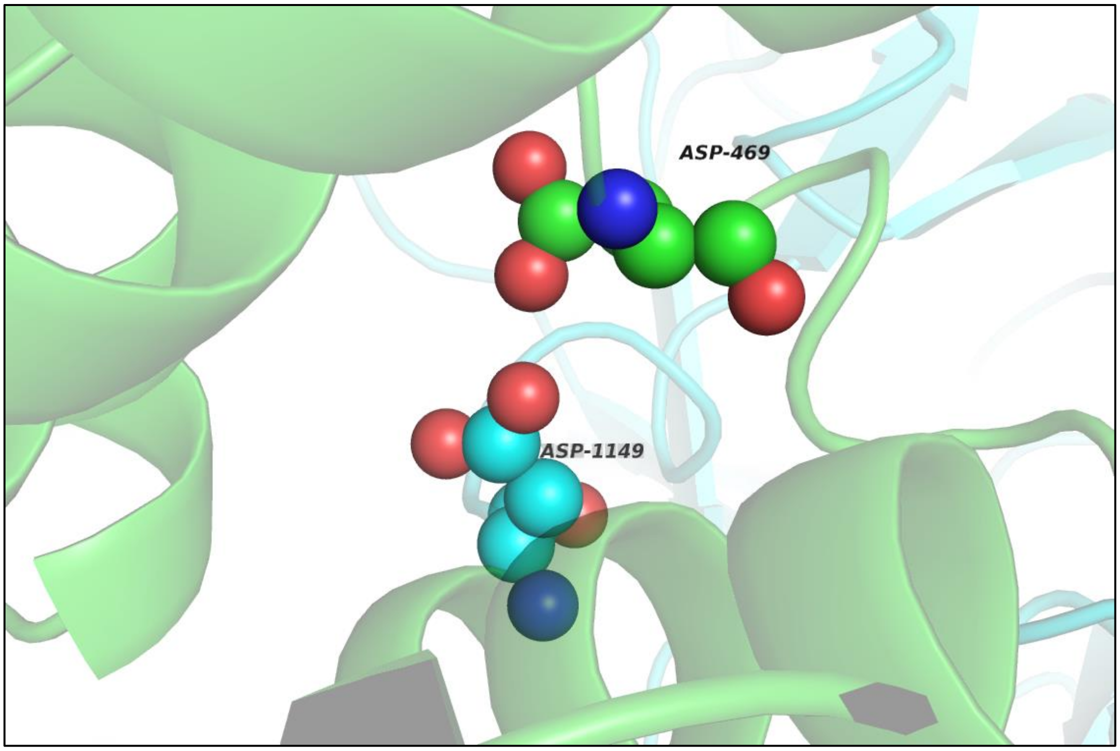

1. BoNT/E:D469 – NTNHE:D11492

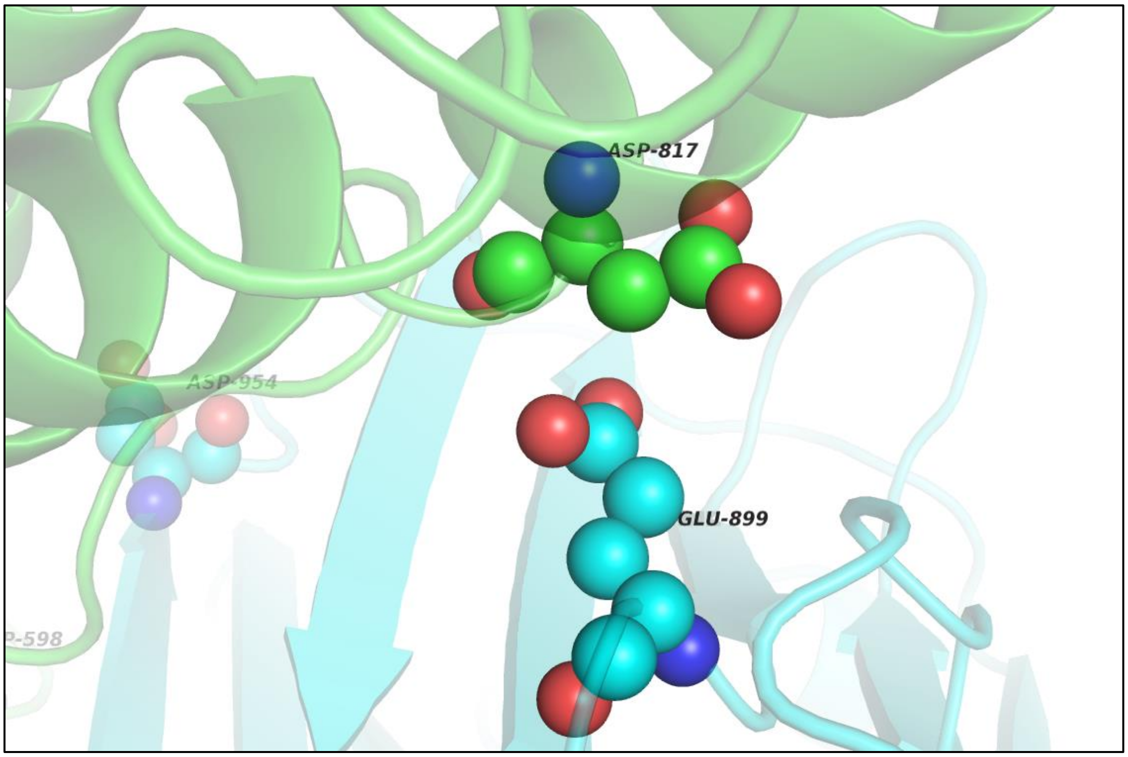

BoNT/E:E810 – NTNHE:E899

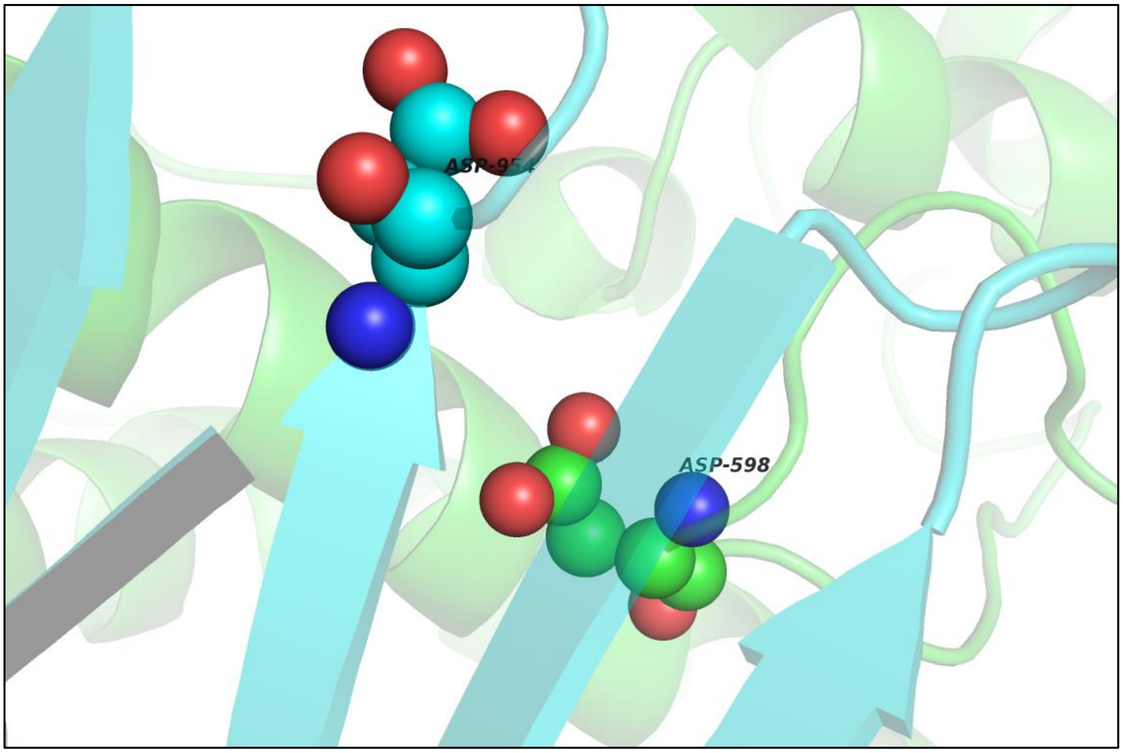

3. BoNT/E:D598 – NTNHE:D954;

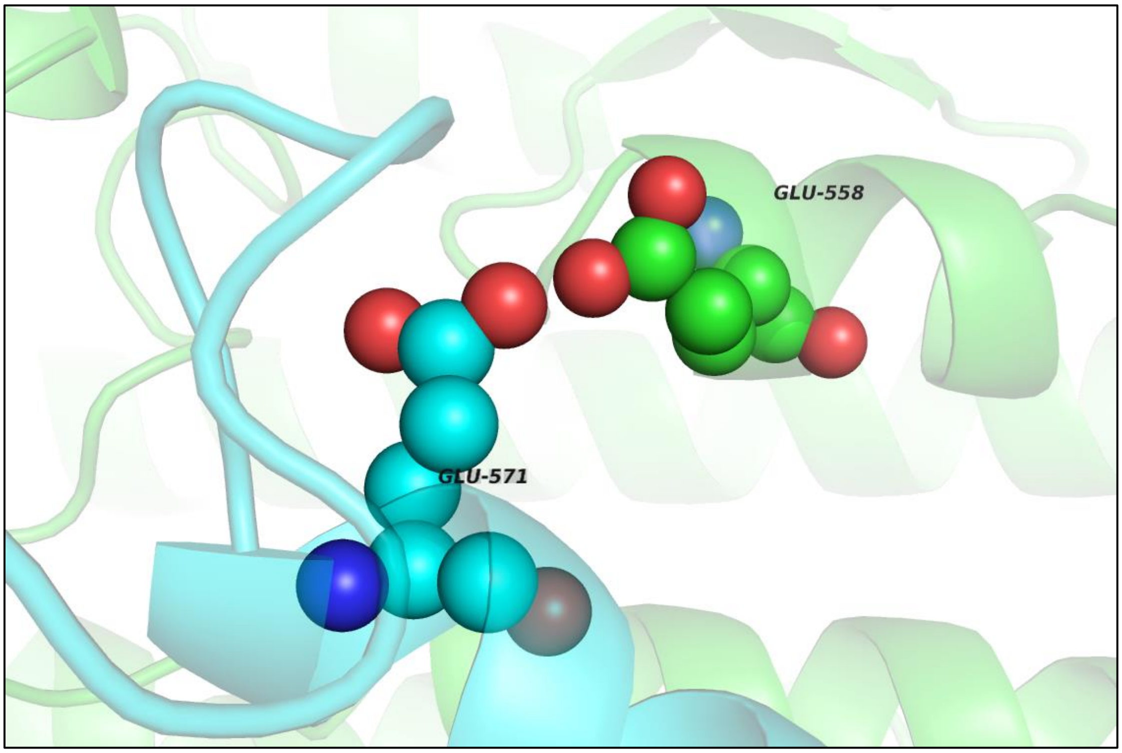

4. BoNT/E:E558 – NTNHE:E571

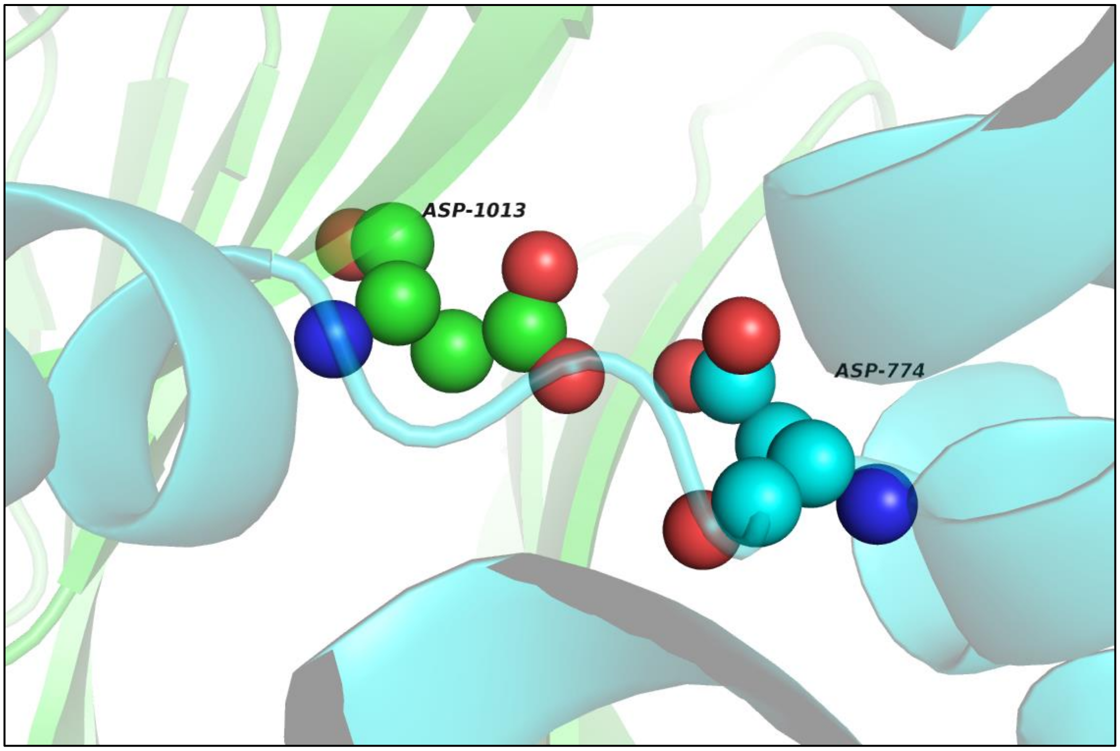

5. BoNT/E:D1013 – NTNHE:D774

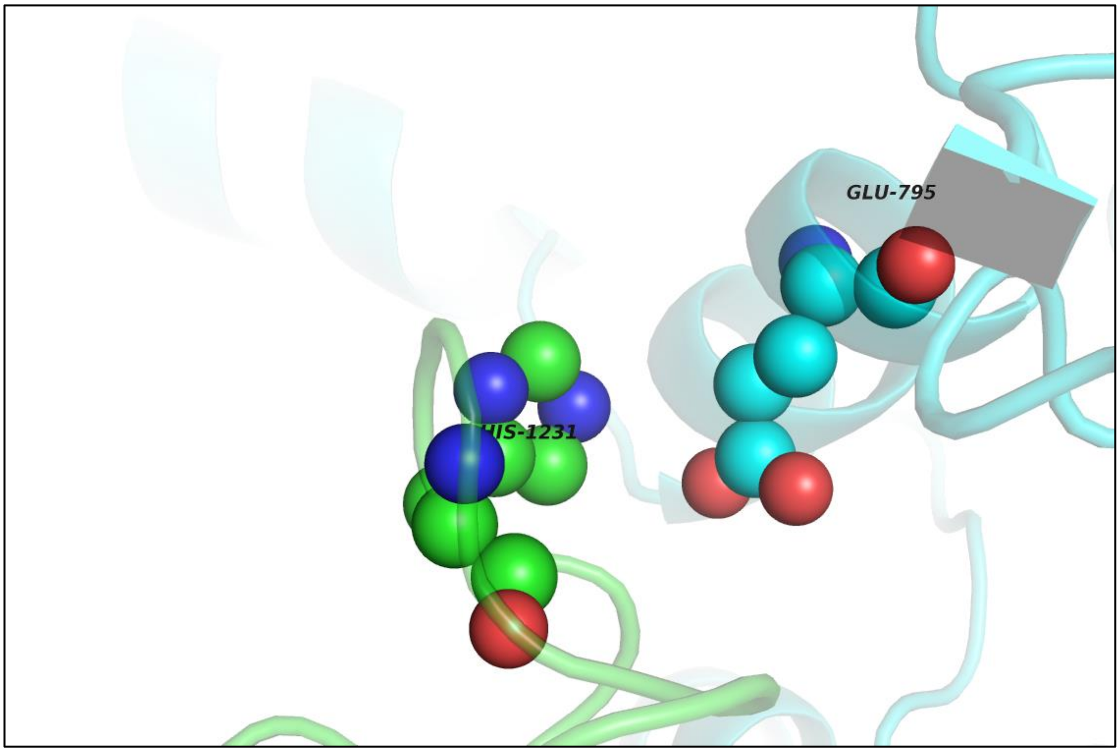

6. BoNT/E:H1231 – NTNHE:E571.

Figure S6. The six acidic clusters and their corresponding electrostatic potential at the interface. BoNT/E and NTNHE are shown in ribbons representation in green and cyan. The acidic residues are shown in sphere model with their carbon atoms in green and cyan, respectively. The interface at each of these clusters displays negative electrostatic potential.

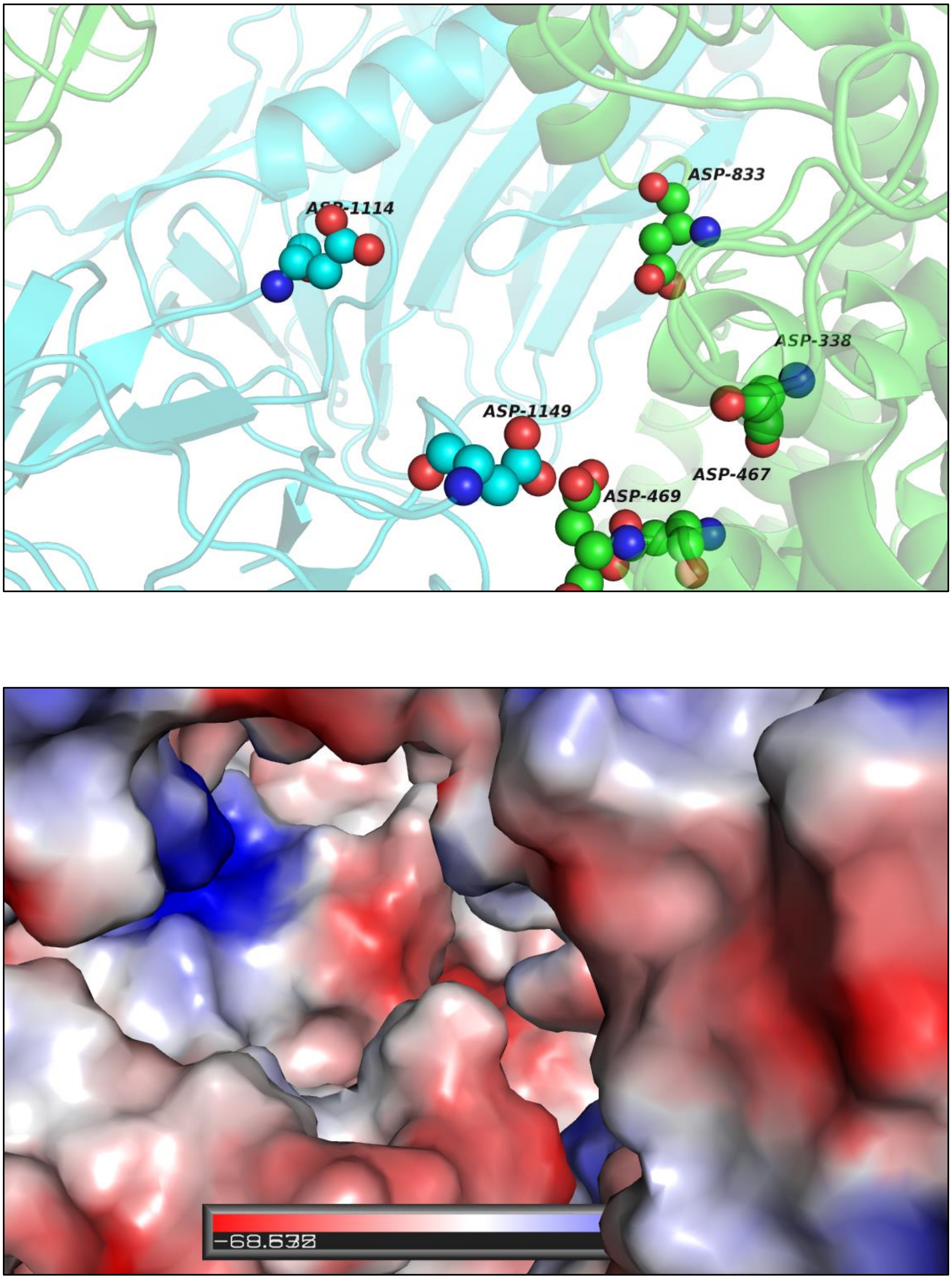

Cluster 1: BoNT/E: D338, D467, D469 and D833. NTNHE: D1149 and D1114

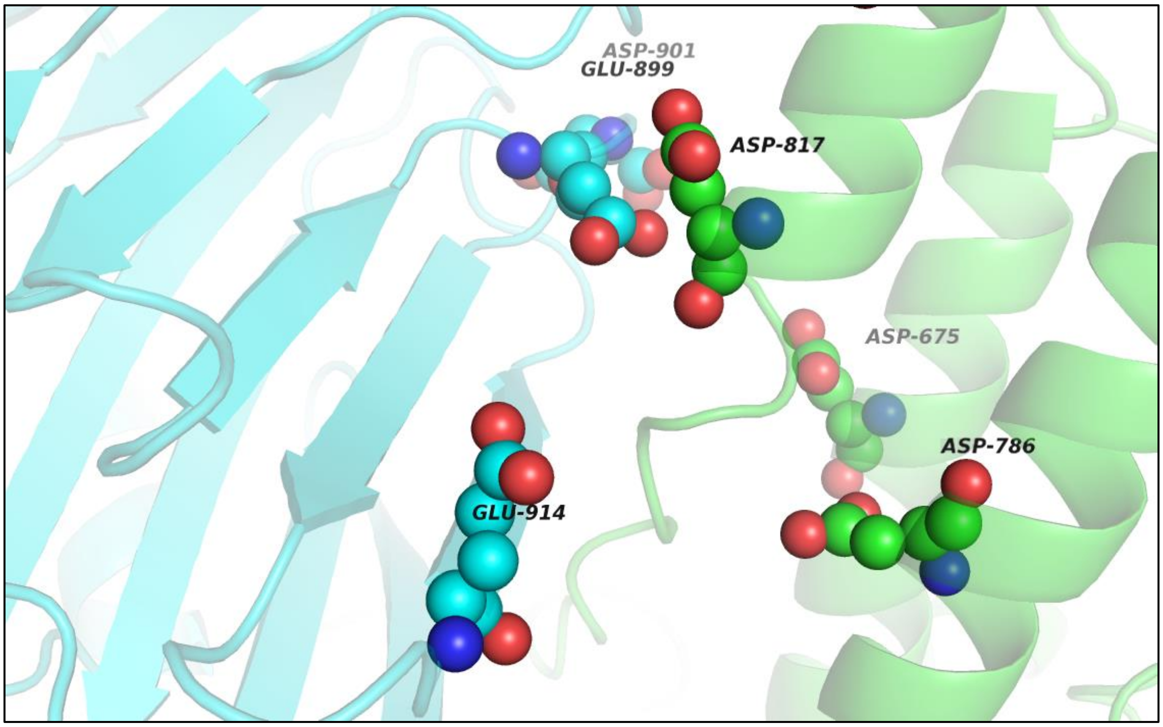

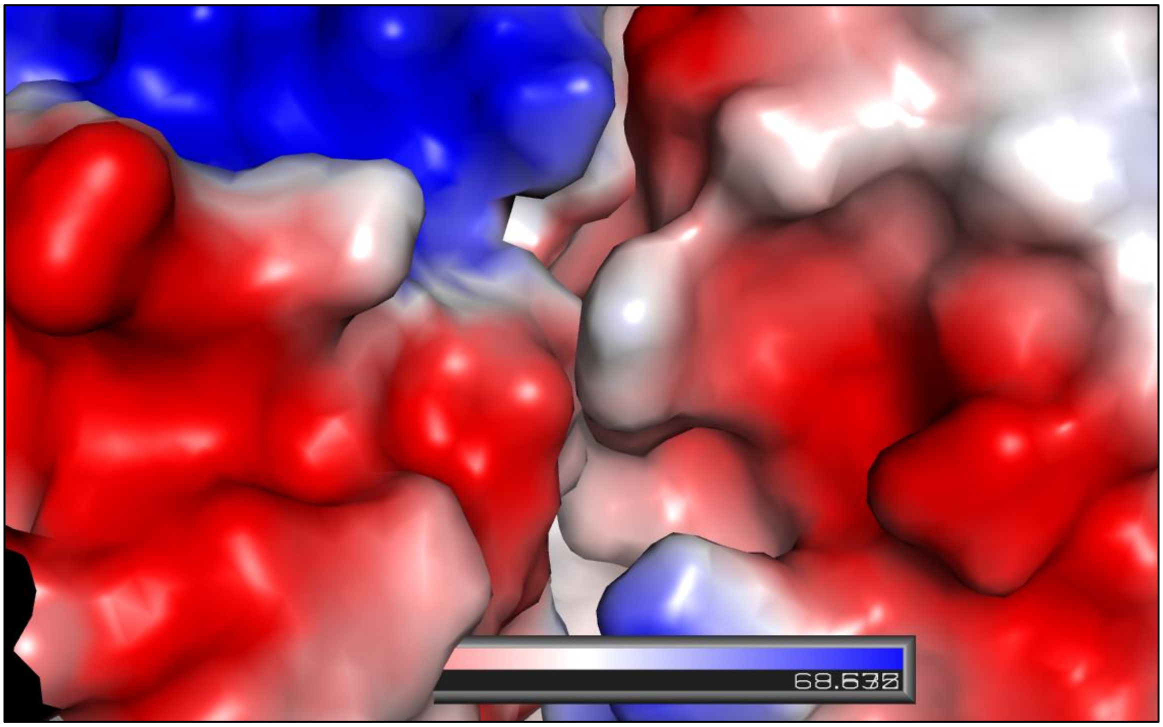

Cluster 2: BoNT/E: E810, D786, D817 and D675. NTNHE: E899, E914 and E901.

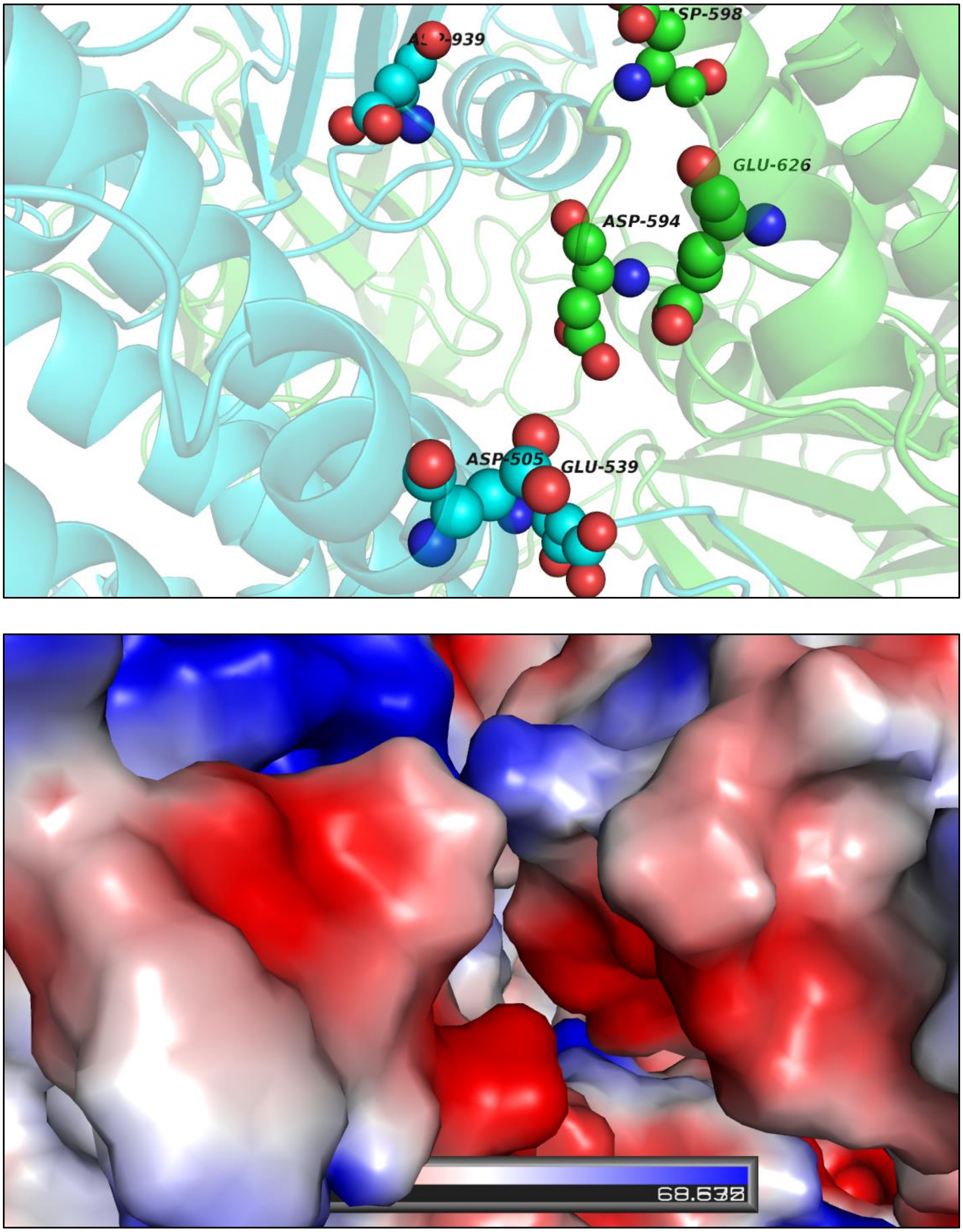

Cluster 3. BoNT/E: D598, D594 and E626. NTNHE: E953, E539, D505, D954 and D939.

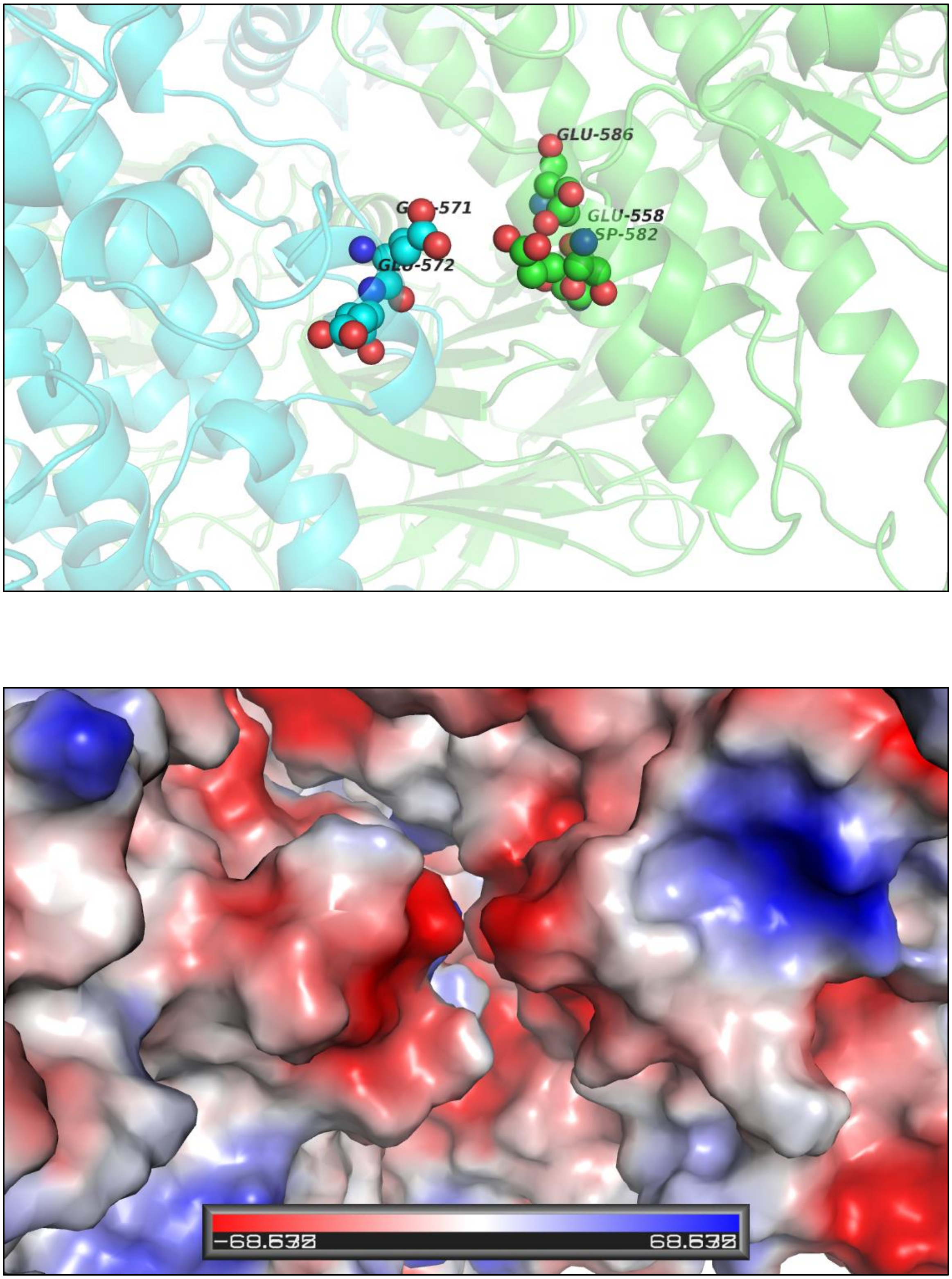

Cluster 4. BoNT/E: E558, E586 and D582. NTNHE: E571 and E572

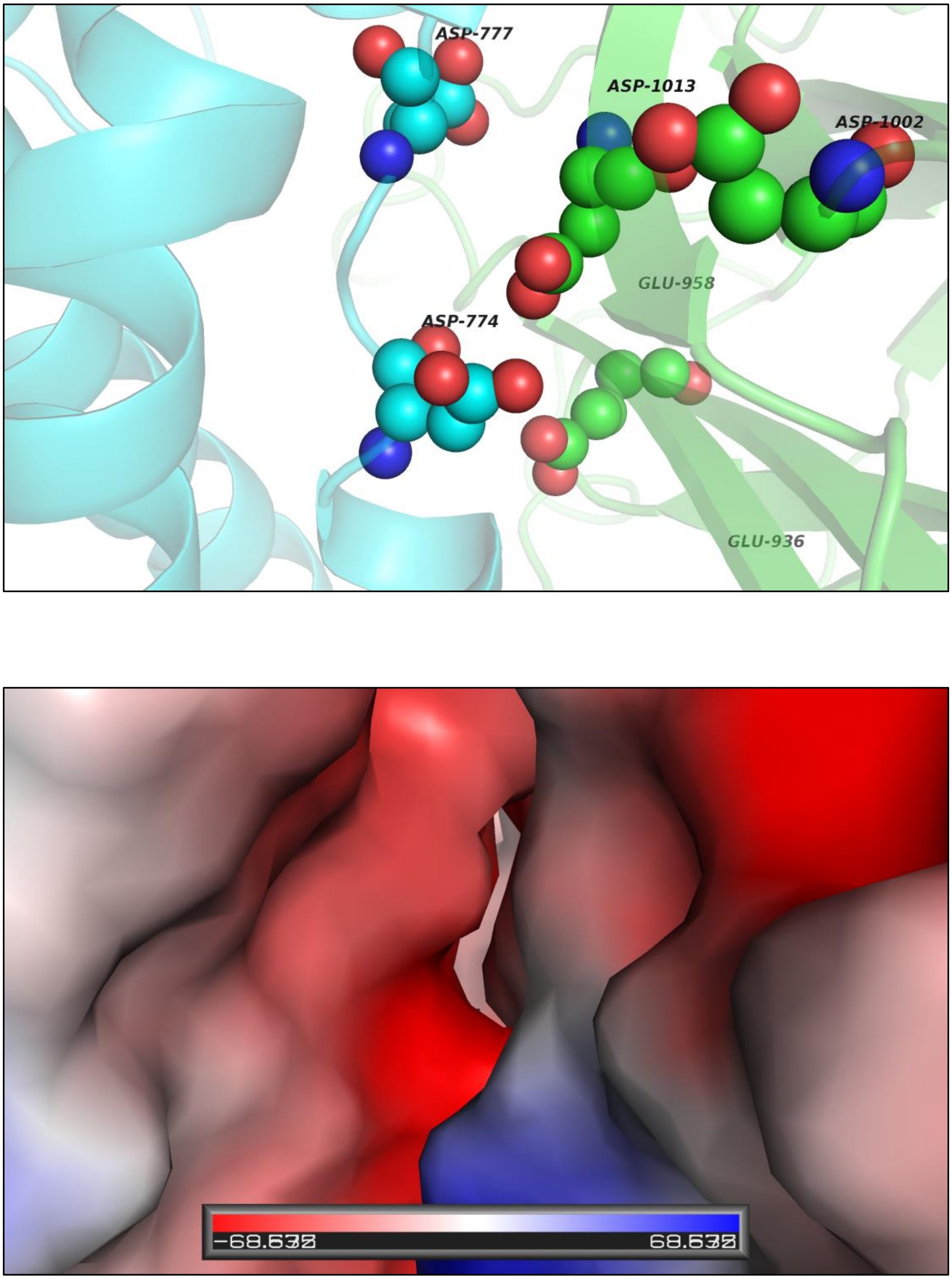

Cluster 5. BoNT/E E936, E958, D1013 and D1002. NTNHE: D774 and D777

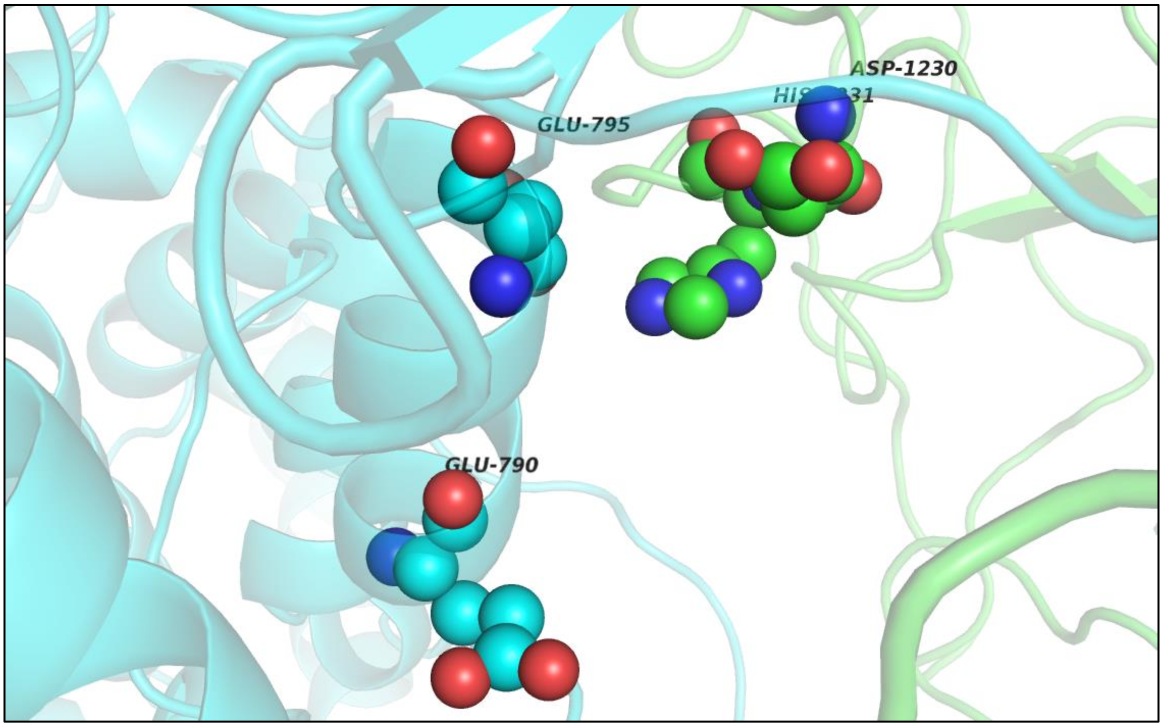

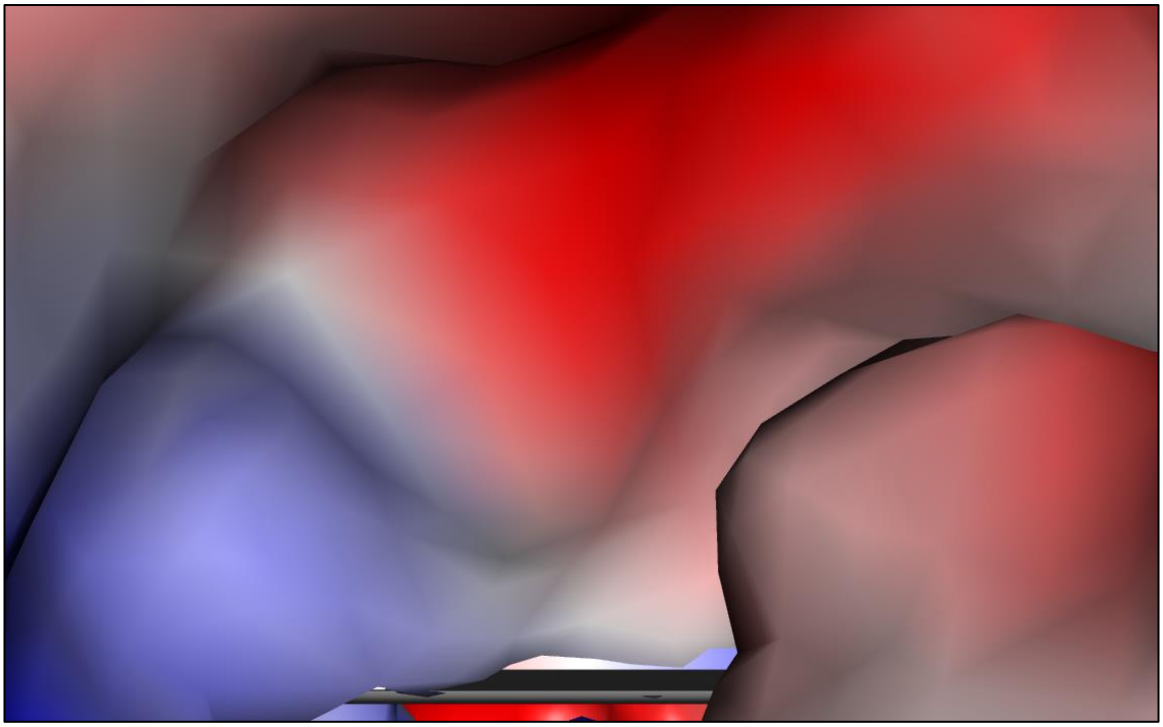

Cluster 6. BoNT/E: H1231 and D1230. NTNHE: E795 and E790.

Fig S7. Superposition of PTC-A(M) and PTC-E(M). Though the conformation of BoNT/A (red) and BoNT/E (green) in the wild type crystal structures are different, they have the same conformation in M complex.

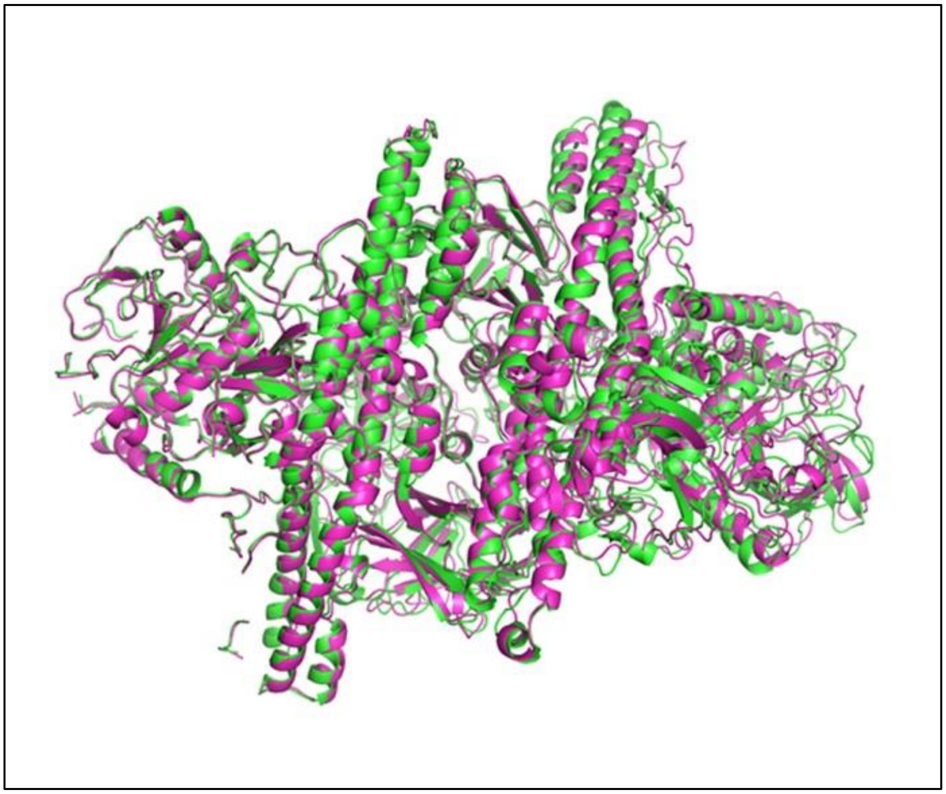

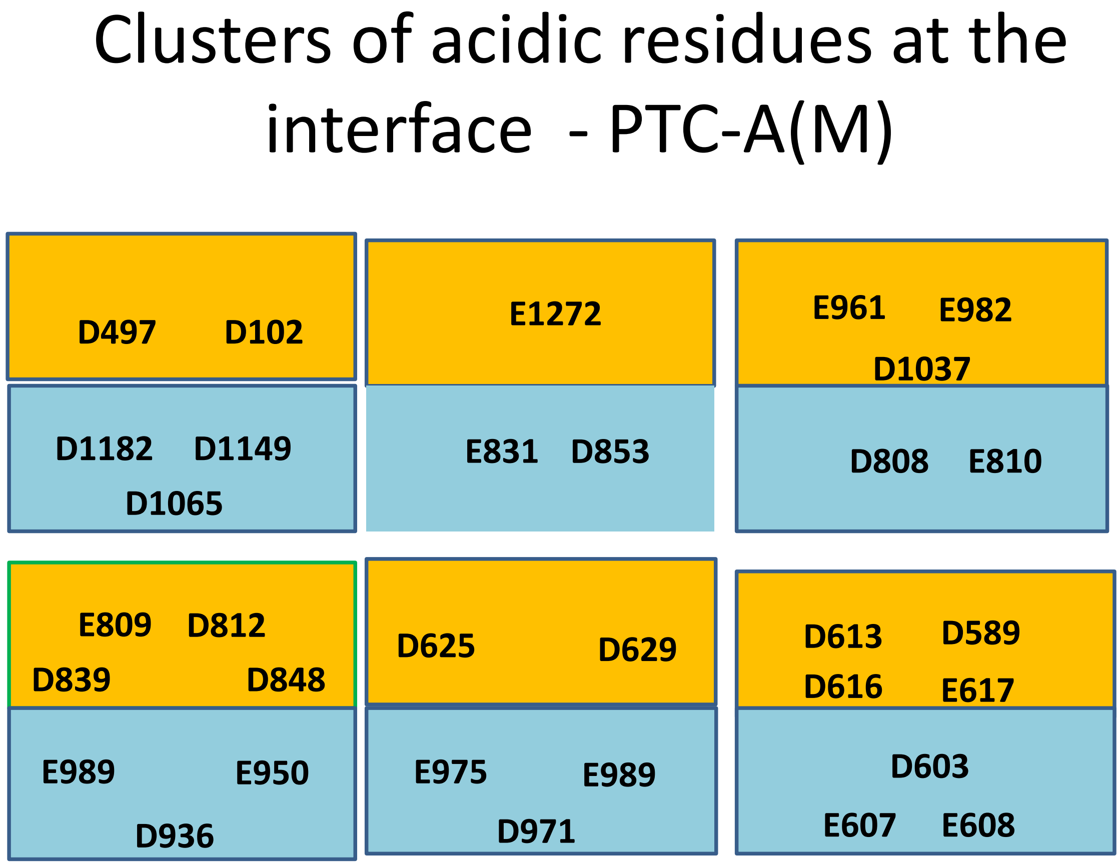

Figure S8. A schematic diagram showing acidic interactions at the interface of PTC-A(M). Six clusters are shown here similar to those in PTC-E(M). Residues of BoNT/A are in orange box in each cluster and residues from NTNHA are shown in light blue box. Coordinates from Pdb id 3V0A were used to prepare this figure.
